# Salt Tolerance Evaluation of Cucumber Germplasm under Sodium Chloride Stress

**DOI:** 10.3390/plants12162927

**Published:** 2023-08-12

**Authors:** Libin Li, Lianda Du, Qiwei Cao, Zonghui Yang, Yihan Liu, Hua Yang, Xi Duan, Zhaojuan Meng

**Affiliations:** 1 Key Laboratory of Greenhouse Vegetable Biology of Shandong Province, Vegetable Science Observation and Experiment Station in Huang—Huai Region of Ministry of Agriculture (Shandong), Shandong Branch of National Vegetable Improvement Center, Vegetable Research Institute, Shandong Academy of Agricultural Sciences, Jinan 250100, Chinacaoqiwei2004@sina.com (Q.C.);; 2 College of Horticulture Science and Engineering, Shandong Agricultural University, Taian 271018, China; 3 College of Horticulture, China Agricultural University, Beijing 100193, China; 4 College of Agricultural Science and Technology, Shandong Agriculture and Engineering University, Jinan 250100, China

**Keywords:** cucumber, NaCl stress, survival rate, cluster analysis, principal component analysis

## Abstract

Cucumber (*Cucumis sativus* L.) is an important horticultural crop worldwide. Sodium (Na^+^) and chloride (Cl^−^) in the surface soil are the major limiting factors in coastal areas of Shandong Province in China. Therefore, to understand the mechanism used by cucumber to adapt to sodium chloride (NaCl), we analyzed the phenotypic and physiological indicators of eighteen cucumber germplasms after three days under 100 and 150 mM NaCl treatment. A cluster analysis revealed that eighteen germplasms could be divided into five groups based on their physiological indicators. The first three groups consisted of seven salt-tolerant and medium salt-tolerant germplasms, including HLT1128h, Zhenni, and MC2065. The two remaining groups consisted of five medium salt-sensitive germplasms, including DM26h and M1-2-h-10, and six salt-sensitive germplasms including M1XT and 228. A principal component analysis revealed that the trend of comprehensive scores was consistent with the segmental cluster analysis and survival rates of cucumber seedlings. Overall, the phenotype, comprehensive survival rate, cluster analysis, and principal component analysis revealed that the salt-tolerant and salt-sensitive germplasms were Zhenni, F11-15, MC2065, M1XT, M1-2-h-10, and DM26h. The results of this study will provide references to identify or screen salt-tolerant cucumber germplasms and lay a foundation for breeding salt-tolerant cucumber varieties.

## 1. Introduction

Salinity is a major abiotic stressor that causes a significant decline in plant growth and biomass production [1,2,3]. Globally, up to one-third of crop yield losses are attributed to salinity [4]. As a result, scientists have long sought to understand and improve the mechanisms of salinity/salt tolerance in crops [5,6]. Screening and cultivating salt-tolerant varieties is one of the effective ways of alleviating salt-stress-induced damage in crops and ensuring the efficient utilization of saline soils [7]. The salt tolerance of crops has also been improved through cross-breeding [8], grafting [9], and treatment with exogenous substances. An example includes the application of phosphate-solubilizing bacteria to corn, which can effectively improve plant growth under lime-induced salinity stress [10]. The screening for salt-tolerant germplasms is important when crops are hybridized for salt tolerance. Chloride and sodium ions are the main components of saline soils and thus form the basis of cucumber salt tolerance research. A complex signal response network system [11] perceiving sodium chloride (NaCl) stress signals allows for saline-tolerant cucumber varieties to grow in saline soils. Plants reduce salt-stress-induced damage by adjusting their metabolite contents or enzyme activity, such as regulating the accumulation of various amino acids and organic acids under salt stress [12]. Moreover, overexpressing chloroplast proteins and ensuring a sufficient supply for plant growth improves photosynthetic activity, which enhances the salt tolerance of the cultivars under salt stress [13].

Cucumber (*Cucumis sativus* L.) is an economically important horticultural crop worldwide [14,15,16] and one of the main vegetable crops with the largest protected cultivation area and widest planting range in China [17]. However, cucumber is salt-sensitive, considerably reducing plant growth, development, yield, and quality in low salt concentration [18,19]. Under NaCl stress, the biochemical indicators such as chlorophyll content and morphological indexes in cucumber seedlings are also altered [20,21,22]. Additionally, the stem thickness and plant height of cucumber seedlings are significantly decreased [23]. NaCl stress also induces membrane peroxidation in cucumber seedlings, which significantly increases the permeability of the cell membrane, damages the cell membrane structure, and increases malondialdehyde (MDA) in leaves [24].

Several methods of evaluating salt tolerance in plants or tissues have been reported, including the use of dry and fresh weights, growth indicators [25], leaf water status [5], and the accumulation of reactive oxygen species (ROS) [26]. The exposure of *Atriplex portulacoides* to 200 mM NaCl resulted in a 30% increase in the whole-plant dry weight compared with the control [27], whereas in cucumber, the growth was reduced and toxic symptoms were apparent under 100 and 150 mM NaCl [20]. In barley, the reduction was earlier in shoots than in roots. The reduction in total chlorophyll may be attributed to a concentration effect of pigments as a consequence of the restriction in growth when exposed to salinity [28].

Although the growth was reduced at high salinity, the plants remained alive and did not exhibit any symptoms of toxicity such as chlorosis, or leaf necrosis. Interestingly, the instantaneous water use efficiency increased significantly [27]. This species can tolerate high NaCl concentrations without showing symptoms of toxicity [29]. However, cucumber readily shows symptoms of toxicity even under low levels of salinity [18]. The symptoms of stress and damage can be detrimental to the survival of plants when soil salinity is just a transient state of stress [29]. Therefore, the salt injury index and survival rate are the best plant tolerance indications under abiotic adversity. They are markers of plant injuries and crucial indicators of salt tolerance [30,31].

Salt stress alters membrane lipid content and lipid biosynthesis pathways in the plasma membrane and tonoplast [23], and the membrane permeability directly reflects the degree of membrane injury; hence, the malondialdehyde (MDA) content indirectly indicates the degree of membrane damage [24]. Proline together with glycine betaine osmotically balance and protect leaves from oxidative stress [29]. Plants protect cells from the adverse effects of ROS using protective enzymes, including superoxide dismutase (SOD), peroxidase (POD), and catalase (CAT), which have strong antioxidant properties. These proteins scavenge ROS, protecting the cells from oxidative damage [32,33], which balances the osmotic potential in plants improving their resistance against salinity [34]. Therefore, these proteins are important indicators of the salt tolerance of cucumber germplasms or varieties.

Salt tolerance is a comprehensive phenomenon, and only one or two indexes are not sufficient to evaluate the salt tolerance of a germplasm; thus, an extensive analysis is required [35,36]. It has been reported that salt-tolerant rice varieties were selected via a comprehensive analysis at the germination stage, whereby the vitality index was identified and used to evaluate the salt tolerance [37]. At present, the comprehensive evaluation of salt tolerance indexes is being widely conducted via principal component analysis [38,39].

In China, cucumber is one of the primary cultivated vegetables planted on a large scale in Shandong Province. However, the soil salinization challenge is becoming more serious in this province, and salt-tolerant germplasm resources are relatively scarce. In this study, eighteen cucumber germplasms were treated with 100 and 150 mM NaCl, which was the same as that used in a previous study on salinity. The aim of this study was to screen out salt-tolerant and salt-sensitive cucumber germplasms by different physiological and biochemical indexes. However, there is no standard evaluation method for salt tolerance. Therefore, we screened out eighteen salt-tolerant cucumber germplasms through a principal component composite score, cluster analysis, and phenotype observation. We hypothesized that salt-tolerant germplasms could be screened under NaCl treatment to aid in later breeding. The findings of this study provide a theoretical basis for the identification and breeding of salt-tolerant cucumber.

## 2. Results

### 2.1. Effects of NaCl Stress on Plant Growth Phenotype of Various Cucumber Seedlings

**Figure 1:**
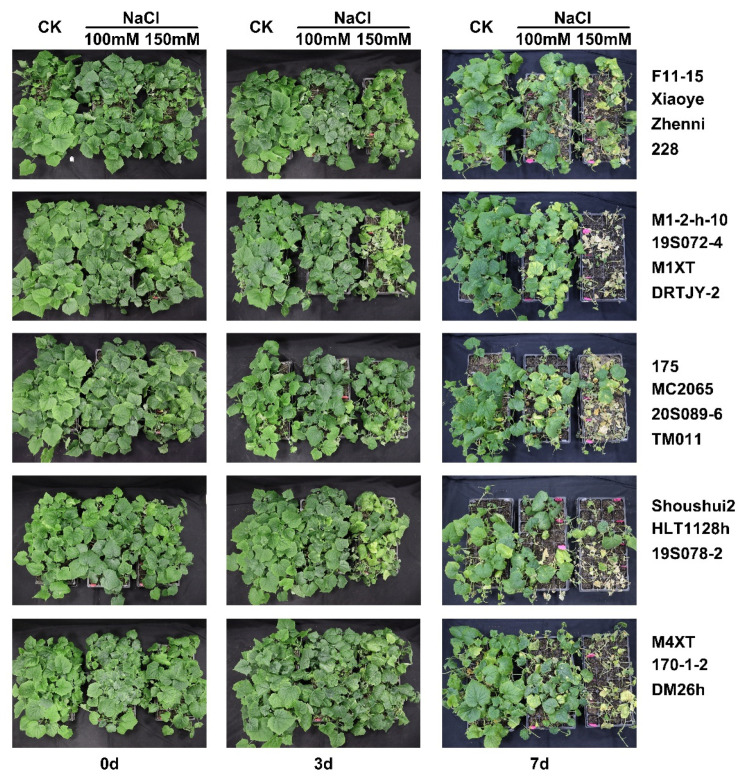
Growth phenotype of eighteen cucumber germplasm seedlings under NaCl stress for 0, 3, and 7 days. The three major columns represent processing times of 0, 3, and 7 days, respectively. Each small column represents tap water, 100 and 150 mL NaCl, labeled as CK, 100 mM, and 150 mM. Each row has a germplasm, with the name on the right.

The growth phenotype of eighteen cucumber germplasm seedlings under 100 and 150 mM NaCl stress for 0, 3, and 7 days was shown. Compared to the CK, there were significant differences among the phenotypes of eighteen cucumber germplasms, including yellow–green leaves, wilting of the leaves, and stem lodging three days after treatment with 100 and 150 mM NaCl. Some germplasms, such as M1-2-h-10, 19S072-4, M1XT, TM011, and 175, showed acute changes in leaf color, water loss, or stem lodging. These symptoms became more pronounced after 7 days, and only about a quarter of the germplasm seedlings survived in 150 mM NaCl (Figure 1). F11-15, Zhenni, MC2065, Shoushui2, and HLT1128h had low damage, and were tolerant to salt.

### 2.2. Effects of NaCl Stress on Salt Injury Index and Survival Rate of Various Cucumber Seedlings

The salt damage degree and index across the eighteen cucumber varieties increased as the NaCl concentration rose. In 100 mM NaCl, more than half of the 19S072-4 and 19S078-2 were damaged, while they recorded the same damage index of 25% (Table 1). In 150 mM NaCl, all the cucumber seedlings across eighteen germplasms displayed salt damage symptoms, among which 19S072-4, M1-2-h-10, TM011, 20S089-6, M4XT, Zhenni, and Xiaoye suffered intense salt damage and were classified as the salt-sensitive group with a salt damage index more than 45%. On the contrary, Shoushui2, HLT1128h, MC2065, and F11-15 had low salt damage, with a salt damage index below 30%, and were classified as the salt-tolerant group.

Under the three NaCl treatments, including 0, 100, and 150 mM NaCl, only the cucumber seedlings treated with 150 mM NaCl died. Therefore, the survival rate of cucumber seedlings at the 150 mM NaCl concentration was used to compare the salt tolerance across the cucumber germplasms. The survival rates of Shoushui2 and HLT1128h seedlings treated with 150 mM NaCl were 100%. On the contrary, the survival rates of 19S072-4, TM011, 20S089-6, DM26h, M4XT and Xiaoye were 9.09, 16.67, 11.11, 8.33, 11.11, and 12.50%, respectively. At the same time, M1-2-h-10, 175 and 170-1-2 were all dead and recorded a 0% survival rate.

### 2.3. Effects of NaCl Stress on Plant Height and Stem Diameter

**Figure 2:**
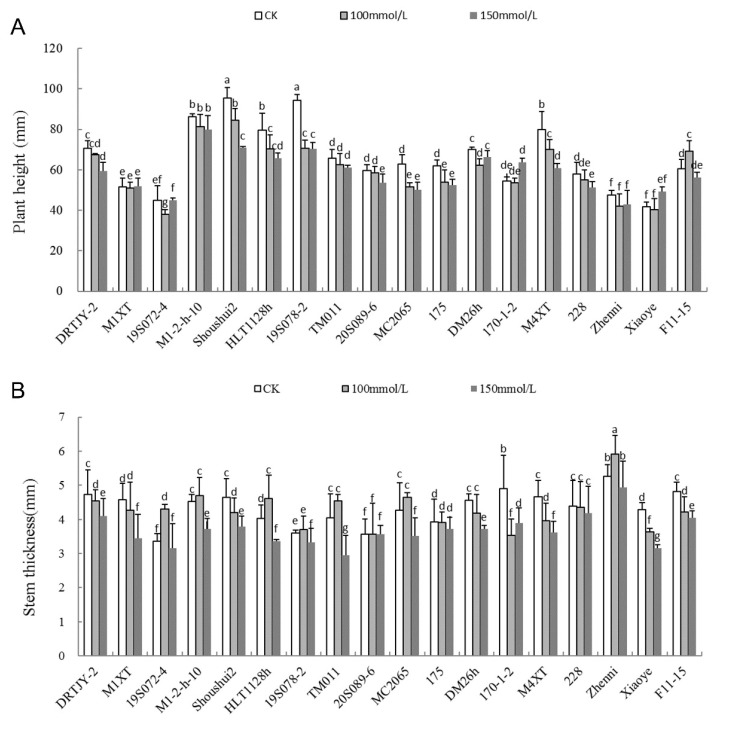
Height (**A**) and stem thickness (**B**) of cucumber seedlings under NaCl stress. Note: The values are the means ± SE (*n* = 3). The bars represent the standard errors. The different lowercase letters indicate significant differences at *p* < 0.05 according to Duncan’s multiple-range test.

Under salt stress, the plant height and stem diameter of cucumber seedlings across eighteen germplasms were inhibited to different degrees. Interestingly, the degree of inhibition increased with the salt concentration and was positively correlated with the salt concentration. Compared with the CK, the plant height of 19S078-2 and MC2065 was significantly inhibited by the 100 mM NaCl treatment, and the inhibition degree was 25.24% and 18.56%, respectively. Additionally, the Shoushui2 plant height was significantly inhibited by 150 mM NaCl and was 24.57 mm-lower than the control, a 25.75% inhibition degree (Figure 2A). The stem diameter of different germplasms recorded a decreasing trend following NaCl treatment. Compared with the CK, 170-1-2 and Xiaoye had the largest decrease in 100 mM NaCl, decreasing by 27.82% and 15.40%, respectively. Thus, the magnitude of change was the smallest under NaCl salt stress (Figure 2B).

### 2.4. Effects of NaCl Stress on Chlorophyll Content

**Figure 3:**
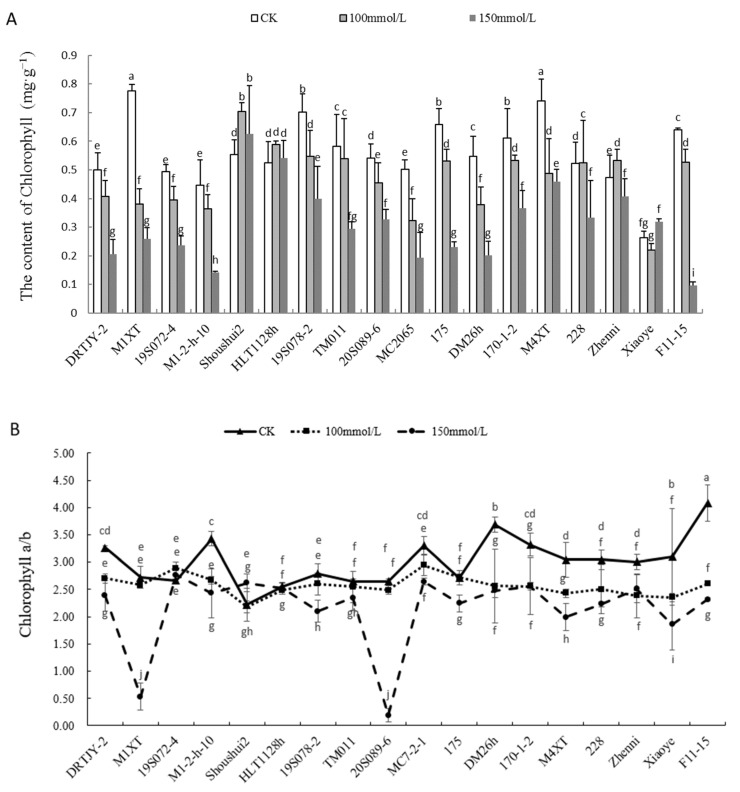
The chlorophyll content (**A**) and *a/b* ratio (**B**) of cucumber seedlings under NaCl stress. Note: The values are the means ± SE (*n* = 3). The bars represent the standard errors. The different lowercase letters indicate significant differences at *p* < 0.05 according to Duncan’s multiple-range test.

The chlorophyll contents and *a/b* ratio in the leaves significantly decreased under 100 and 150 mM NaCl stress in cucumber germplasms, compared to the CK (Figure 3). The chlorophyll contents of cucumber seedlings (except Shoushui2, HTL1128h, TM011, 228, Zhenni, and Xiaoye) were decreased under 100 mM salt stress, and further decreased in all germplasms under 150 mM NaCl except Shoushui2, HTL1128h and Xiaoye. Compared with the control, the chlorophyll content of M1XT, M1-2-h-10, 175, and F11-15 recorded the highest decrease under the 150 mM NaCl treatment, decreasing by 66.63, 69.09, 65.06, and 84.80%, respectively (Figure 3A). On the contrary, 20S089-6, 170-1-2, and 228 recorded the lowest declines in chlorophyll content, decreasing by 39.49, 40.24 and 36.19%, respectively. The chlorophyll *a/b* ratio had roughly the same trend as the contents. The chlorophyll *a/b* ratio significantly decreased in the cucumber germplasms (except 19S072-4) under 150 mM NaCl compared to the CK. 20S089-6 experienced the greatest decline among the germplasms (Figure 3B). We suppose the lower levels of total chlorophyll and chlorophyll *a/b* could be due to decreased content of PSII relative to that of PSI, and high antioxidant enzyme activities.

### 2.5. Effects of NaCl Stress on the Proline Content and POD, SOD, and CAT Activities

**Figure 4:**
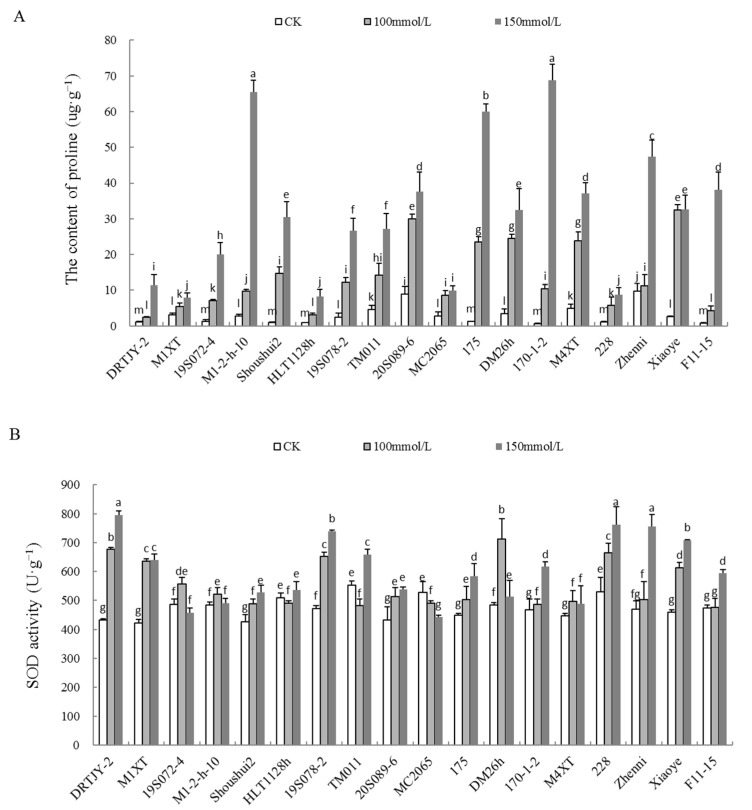

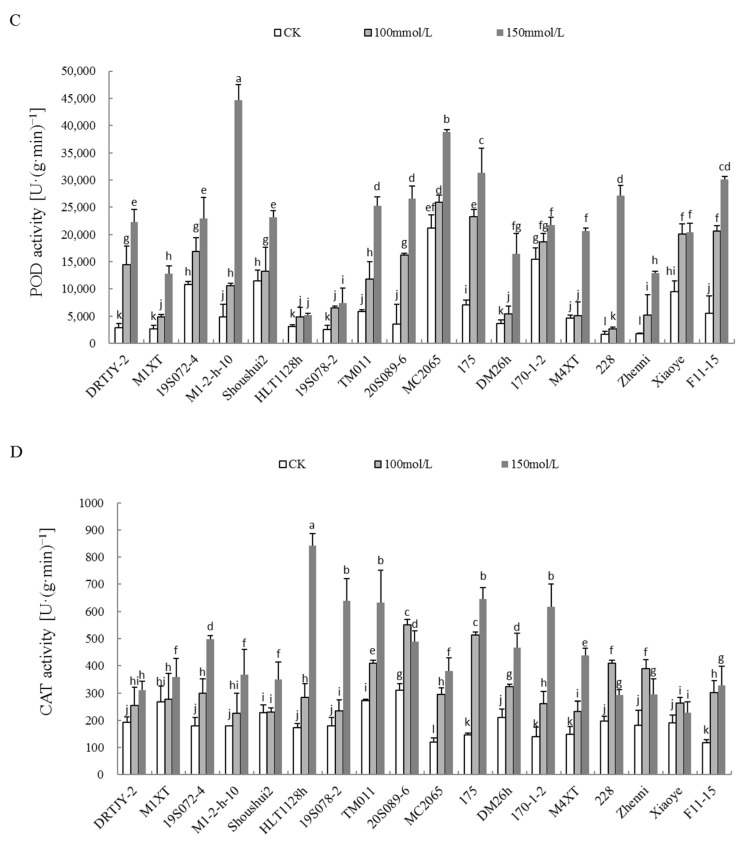
Proline (**A**) and antioxidant enzyme activities (**B**–**D**) in cucumber seedlings under NaCl stress. Note: The values are the means ± SE (*n* = 3). The bars represent the standard errors. The different lowercase letters indicate significant differences at *p* < 0.05 according to Duncan’s multiple-range test.

Compared to the CK, the proline content and SOD, POD, and CAT activities in the cucumber seedlings showed an upward trend, with significant differences at 150 mM (Figure 4).

The content of proline increased with the increasing salt concentration. Compared with the CK, the increase in proline was the most significant under the 150 mM NaCl treatment. Under 150 mM NaCl, the proline content in M1-2-h-10, 175, 170-1-2, and F11-15 was significantly increased by 22.17-, 46.96-, 102.95-, and 44.32-fold, respectively, compared to the control (Figure 4A). Similarly, the SOD activity was significantly increased by 83.65, 51.36, 56.84, 43.63, 60.60, and 54.11% in DRTJY-2, M1XT, 19S078-2, 228, Zhenni, and Xiaoye, respectively (Figure 4B). Compared to the control, the POD activity in M1-2-h-10, 20S089-6, 175, 228, and Zhenni was increased by 8.15-, 6.38-, 3.44-, 15.01-, and 6.23-fold, respectively. (Figure 4C). At the same time, the CAT activity in HLT1128h, 19S078-2, 175, and 170-1-2 was significantly increased by 3.86-, 2.55-, 3.39-, and 3.40-fold, respectively, compared to the control (Figure 4D). The increase in proline content and antioxidant enzyme activities indicated that the germplasms had some ability to resist salt stress, while a lower increase resulted in less resistance to salt.

### 2.6. The Effect of NaCl Stress on the MDA Content of Various Cucumber Seedlings

**Figure 5:**
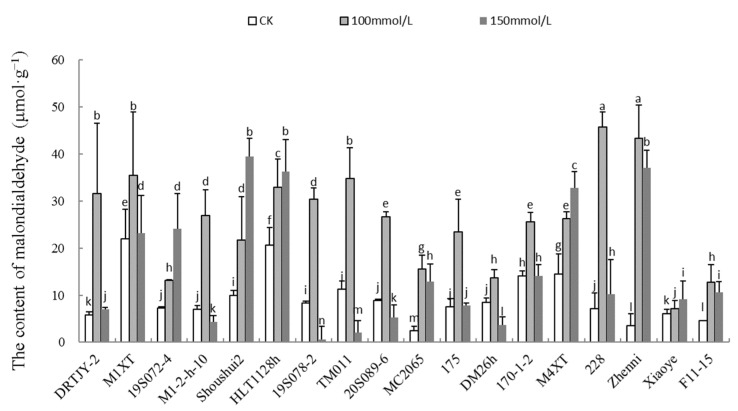
The malondialdehyde content in cucumber seedlings under NaCl stress. Note: The values are the means ± SE (*n* = 3). The bars represent the standard errors. The different lowercase letters indicate significant differences at *p* < 0.05 according to Duncan’s multiple-range test.

In this study, MDA content increased significantly in the 100 and 150 mM NaCl treatment groups compared to the CK. The MDA content in DRTJY-2, 19S078-2, 228, and Zhenni treated with 100 mM NaCl was significantly increased by 442.63, 226.72, 538.76, and 1137.41%, respectively (Figure 5). However, 19S072-4, DM26h, and Xiaoye recorded the lowest increases of 80.36, 61.03, and 17.11%, respectively. The MDA content in Shoushui2 treated with 150 mM NaCl was also significantly increased by 297.53%, compared to the CK. The increase in Xiaoye was the lowest at 51.47%.

### 2.7. Cluster Analysis of Salt Tolerance of Cucumber

**Figure 6:**
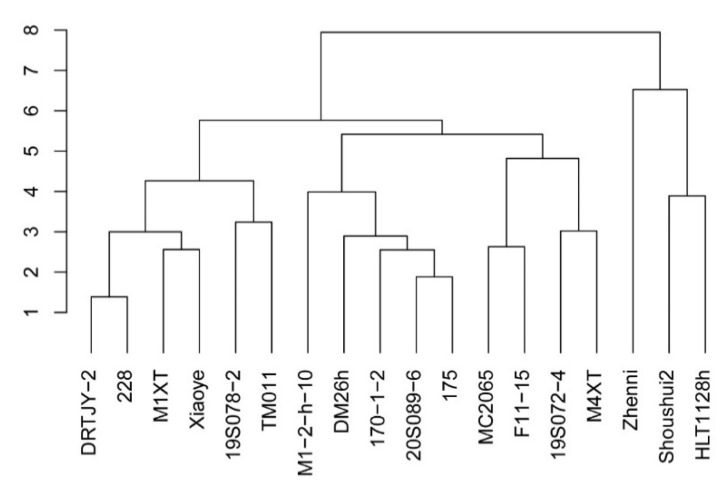
Cucumber germplasm hierarchical clustering.

Based on previous studies, M1-2-h-10 and MC2065 served as references for the classification of salt-sensitive and salt-tolerant germplasms. Cluster analysis divided the eighteen cucumber varieties treated with 150 mM NaCl into three groups based on a high degree of discrimination (Figure 6). The first and second groups consisted of the salt-tolerant germplasms, including HLT1128h, Shoushui2, and Zhenni. The third group was the medium salt-tolerant germplasms, including M4XT, 19S072-4, F11-15, and MC2065. The fourth group was the medium salt-tolerant germplasms, including 175, 20S089-6, 170-1-2, DM26h, and M1-2-h-10. The fifth group had salt-sensitive germplasms, including TM011, 19S078-2, Xiaoye, M1XT, 228, and DRTJY-2.

### 2.8. Principal Component Analysis and Comprehensive Evaluation of Cucumber Salt Tolerance at the Seedling Stage

Ten indexes of plant height, stem diameter, chlorophyll, proline, SOD, POD, CAT activity, salt injury, survival rate, and malondialdehyde were analyzed. The eigenvalues of the first four principal components were greater than 1, with variance contribution rates are 29.39, 19.85, 15.37, and 11.21%, and a cumulative variance contribution rate of 75.82%, implying that the first four principal components reflected most of the ten salt tolerance indicators (Table 2). Hence, the first four principal components were extracted to conduct a comprehensive evaluation and analysis.

**Figure 7:**
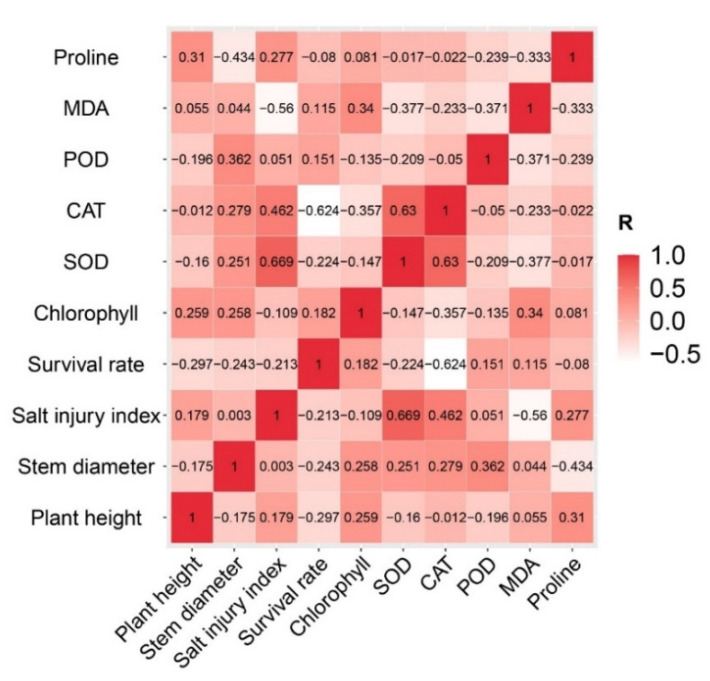
Correlation analysis of the indicators.

Correlation analysis between the ten indicators revealed that several indicators were significantly correlated. For example, the salt injury index was positively correlated with an SOD of 0.669 (Figure 7). This is why the four principal components illustrated the main characteristics. Ranking the comprehensive scores of the cucumber germplasms using the principal component model (Table 3) revealed that the comprehensive score trend of the ten indicators was consistent with the segmental survival rate or cluster results. For example, most of the cucumber germplasms with the top eleven scores had a low survival rate and belonged to the fourth and fifth groups in the cluster analysis. Therefore, salt-tolerant (Zhenni, F11-15, MC2065) and salt-sensitive (M1XT, M1-2-h-10, DM26h) germplasms were finally identified by a comprehensive assessment of the principal component score, phenotype, survival rate, and cluster analysis.

## 3. Materials and Methods

### 3.1. Plant Materials

Eighteen cultivated cucumber germplasms were screened for salt tolerance, including twelve North China types, one South China type, four Eurasian types, and one Japanese Long germplasm (Table 4). The seeds were all provided by Shandong Academy of Agricultural Sciences. Forty-eight seeds were planted per germplasm. A total of eight hundred and sixty-four seeds were uniformly planted on a solid substrate in a cultivation room in April 2021. Plants were grown at 28/22 °C for day/night air temperature, 12 h photoperiod, and 60–70% relative humidity. They were added with different salinities (0, 100, and 150 mM NaCl).

### 3.2. NaCl Treatment on Cucumber Seedlings

Eighteen cucumber germplasms were treated with water (CK), 100 and 150 mM NaCl. Briefly, each seedling was watered with 85 mL of water or NaCl at the three-leaf stage over a 3-day interval. The samples collected from the seedlings with water treatment were used as controls. Fresh leaves per germplasm were collected and stored in liquid nitrogen in 50 mL test tubes, with three replicates per germplasm, and stored at −80 °C in the refrigerator, awaiting further analyses.

### 3.3. Classification of the Salt-Tolerance Level of Cucumbers

The salt damage symptoms were assessed as previously described by Yan [40]. The salt damage index was divided into five levels, including Grade 0: normal leaf growth; Grade 1: drying of a small amount of the leaf edge; Grade 2: drying of less than 50% of the leaves; Grade 3: drying of more than 50% of the leaves; Grade 4: death of the entire plant. The salt tolerance level in all of the eighteen varieties was divided into three levels [20]: salt-resistant, with a salt damage index less than or equal to 30%; salt-tolerant, with a salt damage index of 30–70%; and salt-sensitive, with a salt damage index greater than 70%.

### 3.4. Determination of Growth Index

The growth indexes were determined by measuring the plant height and stem diameter. The plant height was determined as the distance between the stem base and the growing point with a ruler. The stem diameter was determined as the position unified in the stem base using an electronic Vernier caliper.

### 3.5. Quantification of Total Chlorophyll

Frozen leaf tissue was ground in ice-cold 80% (*v*/*v*) acetone, as described by Arnon et al. [41]. The chlorophyll content in the leaf samples was then measured in a 15 mL centrifuge tube at 663 and 645 nm. Finally, the total chlorophyll content per cucumber variety was calculated using the equations described by Cao et al. [20]. Ca (mg L^−1^) = (12.71A_663_-2.59A_645_) V/1000W, Cb (mg L^−1^) = (22.88A_645_-−4.67A_663_) V/1000 W. The contents of chlorophyll (mg g^−1^) = CV/1000 W. C, concentration of chlorophyll (mg L^−1^); V, total volume of extract (mL); W, fresh weight of leaves (g). There were three repetitions in each treatment.

### 3.6. Determination of Proline Content

The proline content in the frozen leaf samples was determined using the acid ninhydrin method, following the specific steps by Li et al. [42]. The 0.1 g leaf samples were placed in 2 mL centrifuge tubes containing 1 mL of 3% sulfosalicylic acid and steel beads. The leaves were ground by shaking, and incubated in boiling water for 10 min. The samples were centrifuged at 10,000× *g* for 10 min after cooling to room temperature. Thereafter, 0.4 mL of the supernatant, 0.4 mL of glacial acetic acid and 0.6 mL of ninhydrin were mixed in a clean centrifuge tube and heated in boiling water for 40 min. After proper shaking, the red substance in the cooled solution from the previous step was extracted in 1 mL of toluene. The absorbance value of the static layering was measured at a wavelength of 520 nm.

### 3.7. Extraction and Assay of Antioxidant Enzymes

Briefly, 0.1 g of frozen leaf tissue was powdered in liquid nitrogen and homogenized in 1 mL potassium phosphate buffer (50 mM; pH 7.0) containing 0.04 g PVP, 5 mM mercaptoethanol, and 0.1 mM EDTA. The homogenates were centrifuged twice at 4500 g for 10 min and 16,000 g for 15 min at 4 °C. The supernatants obtained were for the enzyme activity analyses [43].

SOD, POD, and CAT activities were analyzed as previously described [44] and improved. For SOD activity determination, 25 μL extracting enzyme solution was added to 3 mL of the reaction solution (50 mM phosphate buffer at pH 7.8, 13 mM methionine, 63 μM NBT, 1.3 μM riboflavin, and 1.0 mM EDTA) in a test tube under dim light. The reaction was conducted at 5000 lux illumination for 10 min at 25 °C. Finally, the solution absorbance of the solution was measured at 560 nm wavelength. For POD activity assessment, 2.9 mL of the reaction mixture (50 mM phosphate buffer (pH 7.0), 0.1 mM EDTA, 30 mM H_2_O_2_, and 100 mM Guaiacol) was added to 100 μL of crude extract. The formation of tetra-guaiacol was measured at 470 nm. For CAT activity, 200 μL of the extracting enzyme solution was diluted with 3.2 mL of reaction solution (25 mM phosphate buffer at pH 7.0, 0.1 mM EDTA), 200 μL H_2_O_2_ (10 mM), and 200 μL ascorbic acid (0.25 mM)) in test tubes. Finally, the solution absorbances were measured at 240 nm wavelength for 3 min [45].

### 3.8. Determination of MDA Content

The thiobarbituric acid method determined the MDA content with reference to Heath and Packer [46] and some modifications. Leaf samples (0.1 g) were ground in 1 mL of 5% trichloroacetic acid, and the resulting homogenate was centrifuged at 3000 r/min for 10 min. The supernatant was collected and mixed with 0.4 mL of 0.67% thiobarbituric acid (TBA) and the mixture was incubated in a boiling water bath for 15 min. After cooling, the mixture was centrifuged again for 10 min. The absorbance values of the supernatant were measured at 450 nm, 532 nm, and 600 nm, with distilled water and 0.67% of TBA as the control. The malondialdehyde content was then calculated using the formula: C = 6.45(A_532_-A_600_)-0.56A_450_, where A_450_, A_532_, and A_600_ represent the absorbance values at 450 nm, 532 nm and 600 nm, respectively, and C is the concentration of malondialdehyde (μmol/L). The leaf malondialdehyde content was calculated: W = CV/m, where W is the content of malondialdehyde (μmol/g), V is the total volume of the extraction solution (L), and m is the fresh weight of the leaves (g).

### 3.9. Data Analysis

The cucumber physiological and biochemical indexes were systematically analyzed by cluster analysis. Microsoft Excel 2007 was used to make tables and plots, and SPSS 22.0 was used for significance analysis and principal component analysis. The calculation for the principal component analysis referred to the method of Wang et al. [47]. Comprehensive evaluation of salt tolerance: F(X_j_) = a_1j_X_1j_ + a_2j_X_2j_ + …+a_ij_X_ij_ i = 1, 2, … n; j = 1, 2, … n. Note: F(Xj) is the composite index value of j; a_ij_ is the eigenvalue of a single index corresponding to the eigenvector; X_ij_ is the standardized value of a single indicator.

## 4. Discussion

Salinity is a major abiotic stressor that restricts crop growth and development. In this study, cucumber seedlings were treated with NaCl, followed by screening out the relevant indicators of salt tolerance, which accurately reflected the salt tolerance of cucumber germplasms and can be used to restore the saline soil environment in the coastal areas. Generally, the 100 and 150 mM NaCl concentrations efficiently screened and identified cucumber NaCl tolerance levels [48,49,50], consistent with the research results of Wang et al. [51]. Previous studies revealed that 2 and 4 g·kg^−1^ NaCl inhibited growth and accelerated leaf senescence in *Taraxacum officinale* F. H. Wigg. At 4 g·kg^−1^ NaCl, the leaves were chlorotic and necrotic [52]. However, some plants adapt to salt stress. For example, the leaf intracellular traits of *P. euphratica* coordinate with the leaf economic and hydraulic traits, forming a defense mechanism that reduces salt damage and guarantees growth under saline conditions [53]. Plants can also adapt to salt stress by regulating their hormone levels [54]. The growth of *A. portulacoides* was significantly stimulated at 200 mM NaCl, but reduced at higher levels of salinity [27]. However, in this study, cucumber plant height and stem thickness were significantly inhibited by 150 mM NaCl. Only some germplasms showed no obvious inhibition, such as Zhenni and 19S072-4 (Figure 2). This should be related to the salt resistance of the plants themselves.

The overall morphological indices of the plants under salt stress were significantly inhibited, resulting in leaf wilting or even death [55,56]. Some plants remained alive and did not exhibit any symptoms of toxicity such as chlorosis, leaf necrosis, and leaf water content reduction, even if their growth was reduced [27]. This species is able to tolerate high NaCl concentrations without showing symptoms of toxicity [29]. The phenotypic changes under salt stress are usually determined by the salt injury index. Thus, the salt damage index can reflect the degree of salt damage to plants. Consequently, the salt damage index has been used to select salt-tolerant germplasms in pumpkin [57]. In addition to the salt damage index, hydrogen peroxide and malondialdehyde contents have also been used as important indexes to evaluate the salt tolerance of birch [58]. Generally, the degree of salt-damage increases with the salt damage index under salt stress; thus, the higher the salt damage index, the lower the salt tolerance. The salt damage index is divided into five levels, including normal leaf growth, drying of a small amount of the leaf edge, drying of less than 50% of the leaves, drying of more than 50% of the leaves, and entire plant death. We found that the leaves of M1-2-h-10 and M1XT plants turned yellow under the 100 mM NaCl treatment, and most of the plants under the 150 mM NaCl treatment died in 7 d (Figure 1). Shoushui2 and HLT1128h had high survival rates, while 175 and 170-1-2 had high salt injury indexes, low survival rates, and poor salt tolerances (Table 1). There was a negative correlation between the salt injury index and survival rate (Figure 7), since the germplasms with a high salt damage index had a lower survival rate. However, MC2065 and Zhenni were found to have high salt injury indices and survival rates under the 150 mM NaCl treatment after 7 d. It has been reported that the typical symptom of salinity injury in plants is growth retardation due to the inhibition of cell elongation [59]. This situation shows that the plant has suffered obvious salt damage but also has strong salt tolerance.

The present study also showed that salt stress significantly reduced the total chlorophyll contents (Figure 3A) and the chlorophyll *a/b* ratios (Figure 3B). The reduced production or increased breakdown of chlorophyll molecules under salinity stress limits photosynthetic activity. Moreover, the toxicity of Na^+^ or salt-induced oxidative damage triggers the disintegration of the chloroplast ultrastructure. Decreased photosynthetic pigment, stomatal conductance, impaired enzyme activity, and reduced photosynthetic activity limit the carbon fixation capacity of plants under salt stress conditions [3]. This could be due to the compensatory relationship between the PSII/PSI ratio and chlorophyll *a/b* ratio [60]. PSII inhibition might have reduced the chlorophyll *a/b* ratio (Figure 3B), resulting in the imbalance of the two photosystems [61]. The photosynthetic pigments and chlorophyll contents decreased significantly in all pumpkin genotypes under salt stress, probably due to a salt-induced increase in the activity of the chlorophyll-degrading enzyme, chlorophyllase. The decrease in chlorophyll content under salinity conditions was also reported by Yasar et al. [59]. This may be attributed to a concentration effect of pigments as a consequence of growth restriction when exposed to salinity [28]. However, the chlorophyll content was maintained in the salt-tolerant genotypes, probably because of the high antioxidant enzyme activities that prevented the degradation of leaf chlorophyll [62]. We suppose that the lower levels of total chlorophyll and chlorophyll *a/b* could be due to the decreased content of PSII relative to that of PSI and high antioxidant enzyme activities.

SOD, CAT, and POD activities were also increased in response to salinity stress in this study. Similarly, Khan et al. [63] reported an increase in the three enzyme activities under 120 and 150 mM NaCl in pearl millet. Additionally, Abdelaleim et al. [64] demonstrated elevated SOD and CAT activities in salt-stressed tomato seedlings. A reduction in proline has been observed in several studies [65,66], which revealed that proline does not participate in salt tolerance using CeO_2_ NPs; rather, plants improve other defense mechanisms, such as photosynthesis and the antioxidant system. For example, Avestan et al. [67] revealed salt tolerance mechanisms in strawberries using SiO_2_ NPs, depicted as improved growth, chlorophyll content and relative water content, and limited proline accumulation. In the case of salt-stressed grape plants, using Fe NPs and potassium silicate together mitigated salt injury by lowering MDA, proline, and increasing total proteins and SOD, APX, and POX activities [68]. Herein, the proline content and the antioxidant enzyme activity in cucumber seedlings increased with the salt concentration (Figure 4A–D). Among the cucumber germplasms, DRTJY-2, M1-2-h-10, and HLT1128h had the most obvious increased SOD, POD, and CAT activities, respectively. Studies have revealed that increased antioxidant enzymes imply improved salt tolerance [69,70,71]. MDA is an important stress index in plants [72,73,74]. MDA content increases significantly under abiotic stress due to MDA being closely related to cellular membrane damage. However, it is a member of the HSF family that causes the decrease in plant cold tolerance by increasing MDA content [19]. In this study, Xiaoye had a minimal increase in MDA content under 150 mM NaCl treatment, compared to the CK. Although the MDA content to some extent reflects the tolerance of plants to stress, it was not consistent with the phenotype and survival rate. Generally, a single index cannot fully reflect the cucumber salt tolerance.

Based on this, a principal component composite score model was constructed herein to comprehensively evaluate the salt tolerance of the cucumber germplasm. The principal component composite score model can effectively distinguish between the medicinal values of mugwort and lavender [75]. Principal component analysis has also analyzed and distinctly identified three Chinese herbal medicines and the roots of *Angelica pubescens*, *Codonopsis pilosula*, and *Ligusticum wallichii* with different origins [76]. Additionally, the Tea-DTC model has screened the germplasm resources of drought-tolerant tea trees [77]. The membership function value in wheat selected the best salt-tolerant germplasms [78]. The principal component composite score model has high confidence. However, we found that the results showed some differences among the principal component composite score, cluster analysis and phenotype observation, caused by different calculation methods. Currently, there is no complete system to screen for salt-tolerant germplasms in cucumber, and there are some differences in various screening methods. Therefore, they were combined to provide a more accurate result in salt tolerance research. Finally, six salt-tolerant and salt-sensitive germplasms were selected by a comprehensive evaluation of different physiological and biochemical indices.

## 5. Conclusions

NaCl adversely affects the growth and development of cucumbers. Generally, salt-tolerant germplasms are selected using phenotypic, physiological, and biochemical indicators. This study identified five salt-tolerant and salt-sensitive cucumber groups by a cluster analysis based on ten indicators, including the survival rate, and the salt injury index in cucumber seedlings. In addition, the trend of comprehensive scores was consistent with the segmental cluster analysis and survival rate trends of cucumber seedlings. Thus, by combining the analysis of phenotype, survival rate, cluster, and comprehensive scores, six salt-tolerant and salt-sensitive germplasms were finally identified.

## Figures and Tables

**Table 1:** The salt injury index and survival rate of cucumber seedlings under NaCl stress.

Germplasm	Salt Treatment Concentration/(mmol·L^−1^)	Salt Damage Index/(%)	Survival Rate/(%)
JY-2	100	10.00	
	150	40.00	27.27
M1XT	100	0.00	
	150	35.00	20.00
19S072-4	100	25.00	
	150	70.00	9.09
M1-2-h-10	100	5.00	
	150	50.00	0.00
Shoushui2	100	0.00	
	150	15.00	100.00
HLT1128h	100	5.00	
	150	15.00	100.00
19S078-2	100	25.00	
	150	35.00	23.08
TM011	100	5.00	
	150	70.00	16.67
20S098-6	100	10.00	
	150	45.00	11.11
MC2065	100	10.00	
	150	20.00	70.00
175	100	5.00	
	150	40.00	0.00
DM26h	100	0.00	
	150	30.00	8.33
170-1-2	100	5.00	
	150	35.00	0.00
M4XT	100	5.00	
	150	50.00	11.11
228	100	15.00	
	150	40.00	22.22
Zhenni	100	15.00	
	150	50.00	77.78
Xiaoye	100	5.00	
	150	55.00	12.50
F11-15	100	0.00	
	150	20.00	66.67

**Table 2:** Characteristic value and contribution rate of each component of cucumber seedlings.

Main Ingredient	Eigenvalues	Variance Contribution Rate/(%)	Cumulative Contribution Rate/(%)
1	2.939	29.386	29.386
2	1.985	19.852	49.237
3	1.537	15.372	64.609
4	1.121	11.211	75.820
5	1.071	10.706	86.526
6	0.562	5.623	92.150
7	0.320	3.196	95.345
8	0.222	2.218	97.564
9	0.174	1.741	99.304
10	0.070	0.696	100.000

**Table 3:** The principal component analysis comprehensive scores of different cucumber varieties.

Germplasm	Overall Ratings	Sort
DRTJY-2	0.22285	2
M1XT	−0.25308	15
19S072-4	−0.35987	17
M1-2-h-10	0.03461	9
Shoushui2	−0.04529	12
HLT1128h	−0.36740	18
19S078-2	0.09732	6
TM011	−0.03740	11
20S089-6	−0.00371	10
MC2065	−0.33354	16
175	0.03754	8
DM26h	−0.08068	13
170-1-2	0.18252	4
M4XT	−0.16228	14
228	0.19509	3
Zhenni	0.67673	1
Xiaoye	0.09631	7
F11-15	0.10027	5

**Table 4:** The cucumber varieties and their sources.

Serial Number	Germplasm Name	Ecotype
1	DRTJY-2	North China type
2	M1XT	North China type
3	19S072-4	North China type
4	M1-2-h-10	South China type
5	Shoushui2	Eurasian type
6	HLT1128h	Eurasian type
7	19S078-2	North China type
8	TM011	Japanese Long
9	20S098-6	North China type
10	MC2065	North China type
11	175	North China type
12	DM26h	North China type
13	170-1-2	North China type
14	M4XT	North China type
15	228	North China type
16	Zhenni	Eurasian type
17	Xiaoye	Eurasian type
18	F11-15	North China type

## Data Availability

All data generated or analyzed during this study are included in this published article.
